# Prediction and analysis of key protein structures of 2019-nCoV

**DOI:** 10.2217/fvl-2020-0020

**Published:** 2020-05-12

**Authors:** Qihao Li, Wen Peng, Yu Ou

**Affiliations:** ^1^School of Life Science & Technology, China Pharmaceutical University, Nanjing, PR China

**Keywords:** 2019-nCoV, back mutation, drug screening, homology alignment, structural analysis

## Abstract

**Aim:** The purpose of this study was to predict and analyze the structure and function of 2019-novel Coronavirus (nCoV) key proteins. **Materials & methods:** We obtained the structure and sequence of proteins from related databases and studied them through multiple sequence alignment, homology modeling, sequence analysis, virtual screening, reverse mutation, protein structure overlap and surface property analysis. **Results & conclusion:** We found no significant changes in envelope protein, membrane protein, nucleocapsid protein and key proteases in open reading frame 1ab, and predicted results of proteins and performed molecular dynamics simulations. Based on the surface properties of spike protein and docking results with angiotensin-converting enzyme 2, we believe that the binding ability of spike protein to angiotensin-converting enzyme 2 may be similar to SARS. These studies will help us in fighting 2019-nCoV.

Coronaviridae are members of single-stranded RNA (ssRNA) viruses, and belong to the Nidovirales in taxonomy. We currently know that they only infect vertebrates and are associated with many diseases of humans and animals. Mature coronavirus (CoV) particles range in diameter of approximately 60–220 nm [1]. The most prominent feature is the morphology of the outer envelope of the virus, with obvious rod-shaped extra-membrane particles. This structure, which resembles the crown of medieval European emperors, is the source of its ‘coronavirus’ name [2].

New CoV pneumonia refers to pneumonia caused by a new CoV (2019-nCoV) infection. Since December 2019, some hospitals successively found multiple cases of unexplained pneumonia with a history of South China Seafood Market exposure in Wuhan city, Hubei province, then the pneumonia was confirmed as an acute respiratory infectious disease caused by 2019-nCoV [3,4]. The clinical manifestations are fever, fatigue, dry cough and rare nasal congestion and runny nose. About a half of patients develop dyspnea after 1 week. In severe cases, they rapidly progress to acute respiratory distress syndrome, septic shock, difficult to correct metabolic acidosis and coagulation dysfunction [5]. Up to now, more than 1.5 million patients have been diagnosed and nearly 90,000 people have died. At the same time, fecal-oral and contact transmission was found [6].

2019-nCoV is a sense ssRNA virus that replicates without passing through DNA intermediates. Its genome is the largest of the known RNA viruses, about 30 kb [7,8]. The first two-thirds of the genome can be translated into two large replicase polymers (pp1a and pp1ab). Under the action of PLpro and 3CL protease, pp1a and pp1ab are digested into 16 non-structural proteins (nsp) 1–16, which are assembled to produce multifunctional and membrane-bound replicase complex. Replicase complexes mediate viral genome replication and structural gene translation. 2019-nCoV contains four structural proteins, namely S protein, M protein, E protein and N protein [9]. Among them, S protein is a virus shell component and also an important factor for virus binding to host cell receptors and virus-induced antibody production or cellular immune response [10,11]. The S protein is composed of two subunits, S1 and S2. S1 is spherical and S2 is rod shaped. They combine with each other by intermolecular forces. S1 can bind to receptors on the surface of host cells. S2 can immobilize the entire S protein on the membrane and mediate the fusion of the viral envelope and the host cell membrane [12]. These proteases and structural proteins are often used as targets for drug design.

## Materials & methods

### Key protein multiple sequence alignments

We searched the complete genomes of Wuhan Seafood Market pneumonia virus (2019-nCoV) and other bat CoV in NCBI (NCBI ID is as follows: 2019-nCoV: NC_045512.2, bat SARS CoV RaTG13: MN996532.1, bat SARS CoV RS672 / 2006: FJ588686.1 bat SARS CoV ZC45: MG772933.1, bat SARS CoV BJ01 AY278488.2, bat SARS CoV ExoN1 FJ882956.1 and bat SARS CoV GZ02 AY390556.1), and downloaded the amino acid sequence of each key protein. Multisequence alignments are analyzed using Clustal Omega in the European Bioinformatics Institute. Clustal Omega is a new multiple sequence alignment program that is used to seed guide trees and Hidden Markov Model profile–profile techniques to generate alignments between three or more sequences.

### Predicting key protein structures using homology modeling

After obtaining the amino acid sequence of each key protein in NCBI, the spatial structure of each protein was predicted using the SWISS-MODEL homology modeling method. We entered the amino acid sequence and selected the protein structure of the amino acid sequence with the highest homology to the input sequence as a template, which has been experimentally determined for protein structure. The designated template protein data bank (PDB) IDs for each key protein are 3CL hydrolase (ID: 2z9j), E protein (ID: 5x29), PLpro (ID: 5tl6) and S protein (ID: 6acd and 6acc). When the sequence homology of each template reached more than 75%, the predicted structure obtained was highly reliable.

### Molecular dynamics simulation

Through Discovery Studio 2016 software, we conducted a molecular dynamics simulation of the predicted key protein structure of the virus. The protein molecules were put into physiological saline solvent environment. In the process of minimization and molecular dynamics simulation, the particle-mesh Ewald is used to deal with long-range electrostatic interactions. All chemical bonds related to hydrogen atoms are fixed using the SHAKE algorithm. The Standard Dynamics Cascade process includes five stages: minimization, minimization 2, heating, equilibration and production. Minimization and minimization 2 is minimized by the 2000-step steepest descent method and the 2000-step conjugate gradient method. The heating step is slowly heating up to 300 K. Next, the system performs the simulation of the balancing step under the ensemble of constant pressure normal pressure and temperature (NPT). Simulation time is set to 200 picosecond (ps) and so on, and 100 conformations are obtained in the production item. For the files generated by the molecular dynamics simulation, the trajectory analysis was carried out. In order to evaluate the structure of the simulation system, we performed the calculation of root mean square deviation.

### Computer aided virtual screening

A full-sequence tripeptide library containing 8000 peptides was constructed, and the PLpro-predicted structure obtained by homology modeling was used to virtually screen the peptide library. The specific operation is using Protonate 3D to protonate the structure of the protein. Energy minimize minimizes the added hydrogen bond energy, specifies the catalytic site in the active pocket as a virtual screening target and finally obtains the best-scoring tripeptide.

### Overlapping alignment of protein spatial structure

By overlapping the predicted 2019-nCoV S protein structure with the template BAT SARS CoV S protein (PDB ID: 6acc) structure, we found that there was a spatial structural difference in the S protein between 2019-nCoV and previous bat SARS CoVs. We used Swiss PDB Viewer (SPDBV) to open the predicted S protein structures of 2019-nCoV (yellow) and bat SARS CoV (blue) at the same time, and the S protein of bat SARS CoV was used as the template. According to the sequence alignment results, the identical amino acid sequence from Ala^930^ to Gln^1040^ was designated as the overlap position. Fit molecules were used for intelligent overlap and finally the overlap result was analyzed.

### Back-mutate study of receptor binding domain of S protein

According to the sequence alignment of S protein and docking results of S protein with ACE2, there are two obvious changes in receptor binding domain (RBD) of S protein: first, three of the six amino acids that interact with ACE2 are mutated; second, a large number of prolines in the proline concentrated region of RBD are replaced. Therefore, we reversed the mutated amino acids or replaced them with other amino acids, then docked with ACE2, and used PDBePISA of European Bioinformatics Institute to analyze the docking results.

## Results

### Alignment of amino acid multiple sequences among the key proteins of 2019-nCoV & other CoVs

**Figure 1.**
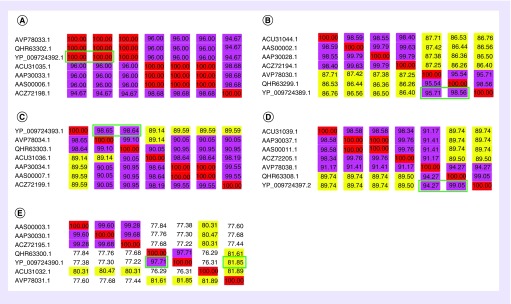
Multiple sequence alignment results of the key proteins among 2019-nCoV and other bat SARS coronaviruses. **(A–E)** represents the amino acid sequence alignment results: envelope protein (E), orf1ab, membrane protein (M), nucleocapsid protein (N) and spike protein (S). Virus code: ACU refers to bat SARS coronavirus (CoV) Rs672/2006, AAP refers to bat SARS CoV BJ01, AAS refers to bat SARS CoV GZ02, ACZ refers to bat SARS CoV ExoN1, AVP refers to bat SARS CoV ZC45, QHR refers to bat SARS CoV RaTG13, and YP refers to 2019-nCoV. The horizontal virus code is consistent with the vertical, and the degree of agreement is expressed as a percentage (red 100%, pink 90–99.9%, yellow 80–89.9%). The green box shows the comparison results of bat SARS CoV RaTG13 and bat SARS CoV ZC45 with higher homology to 2019-nCoV.

We compared homology of 2019-nCoV five key protein structures with other bat SARS CoVs in this section [13]. Five key proteins include open reading frame 1ab (orf1ab) in the nonstructural region and four structural proteins: S protein, E protein, M protein and N protein. The results show that the four main proteins of 2019-nCoV have the highest homology with that of bat SARS CoV RaTG13 and bat SARS CoV ZC45. E protein of 2019-nCoV has 100% homology with that of both bat SARS CoVs (Figure 1A), and the identity of orf1ab, M and N proteins between 2019-nCoV and bat SARS CoVs has also been reached at 94.27% or more (Figure 1B–D). The comparison results of the S protein showed that it still has the highest homology with bat SARS CoV RaTG13 and bat SARS CoV ZC45 reached 97.71 and 81.85%, respectively (Figure 1E). Overall, 2019-nCoV is basically developed from bat SARS CoV, with the highest degree of homology to bat SARS CoV RaTG13, which is consistent with the results of existing studies [14], and the degree of agreement with other SARS CoVs is also greater than 75%, suggesting that we can use the homology modeling method to predict 2019-nCoV protein structure, which can be used for virtual screening, molecular docking and drug design [15,16] for accelerating the development of anti-2019-nCoV drugs.

### Key protein structures of 2019-nCoV are predicted with homologous modeling

**Figure 2.**
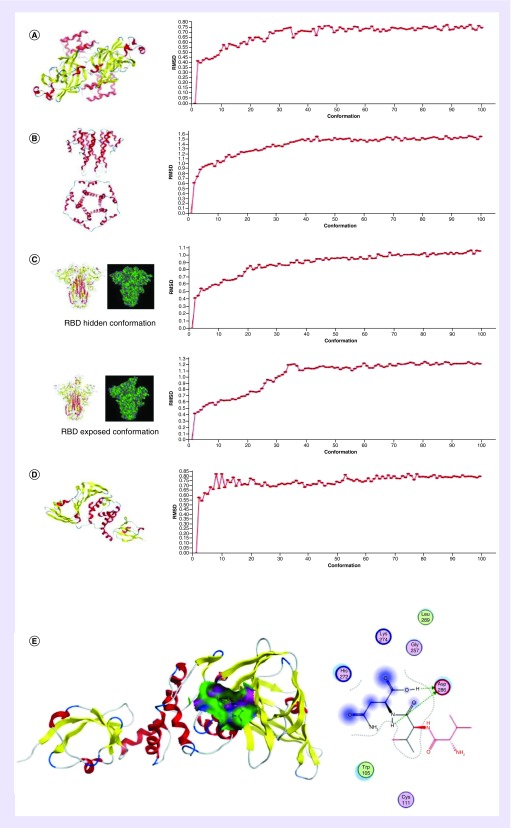
Structure prediction of each key protein and a peptide drug screening results. **(A)** 3CL hydrolase homology modeling prediction structure and RMSD of molecular dynamics simulation. **(B)** E protein homology modeling prediction structure and RMSD of molecular dynamics simulation. **(C)** Two homology modeling prediction structure and RMSD of molecular dynamics simulation of spike protein. **(D)** PLpro homology modeling prediction structure and RMSD of molecular dynamics simulation. **(E)** Tripeptide TP8 interacts with PLpro. TP8 can contact and form hydrogen bonds with the catalytic sites His^272^ and Asp^286^ of the PLpro. RBD: Receptor binding domain; RMSD: Root mean square deviation.

We selected the existing crystal structure of PDB, which is more than 75% consistent with 2019-nCoV’s PLpro, 3CL hydrolase, structural protein S and E, and used SWISS-MODEL’s homology modeling method to predict the structure of each protein of 2019-nCoV [17], as shown in Figure 2A–D. Among them, 3CL protease is the main protein processing enzyme of CoV, which is essential for virus replication and proliferation (Figure 2A) [18]. E protein is also a membrane integrin, consisting of a highly hydrophobic N-terminus (the transmembrane region of E protein) and a C-terminus that extends into the body of the virus (Figure 2B) [19]. The S protein is the main protein that interacts with host cells on the viral coat (Figure 2C).The protein produced by PLpro digestion is necessary for the virus, because it can activate the synthesis of viral mRNA (Figure 2D) [20]. We separately predicted the structure of these key proteins. Ramachandran plot and Profile-3D were used to evaluate the quality of the predicted structure (Supplementary Figure 1), and by using two different S protein templates, we predicted two different states conformation of the S protein during its interaction with ACE2. This is consistent with the research by Wrapp *et al.* [21]. At the same time, we conducted a molecular dynamics simulation, and the root mean square deviation trajectory of each conformation was basically stable, as shown in Figure 2A–D. The structures of these proteins are common targets for drug development. Taking the PLpro structure obtained by homology modeling as a target, and using a peptide with low toxicity and favorable for clinical acceleration as an example [22], the full-sequence library of tripeptides was subjected to screening of antivitual drugs. Finally, the tripeptide with amino acid sequence Val-Val-Asn (TP8) with strong binding ability to ACE2 was obtained. The results show that TP8 can contact and form hydrogen bonds with the catalytic sites His^272^ and Asp^286^ of the PLpro (Figure 2E).

### Differential key protein structure analysis of 2019-nCoV

**Figure 3.**
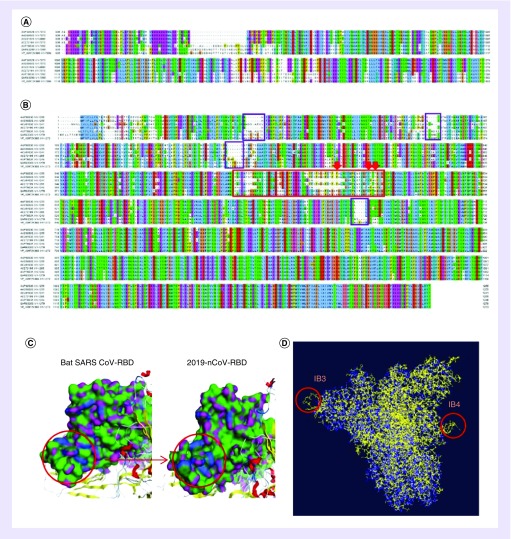
Analysis of structural differences of open reading frame 1ab and spike protein between 2019-nCoV and other bat SARS coronaviruses. **(A)** Orf1ab amino acid multiple sequence alignment results of 2019-nCoV with that of other bat SARS CoV. **(B)** Spike amino acid multiple sequence alignment results 2019-nCoV with that of other bat SARS CoV. In the RBD site, three of the six key amino acid residues (red arrow) that interact with ACE2, and four IBs (1–4; purple box) were inserted into the N-terminus and S2 region of 2019-nCoV. **(C)** Analysis of the surface properties of RBDs of 2019-nCoV and other bat SARS CoVs. The 2019-nCoV polarity becomes more dense after amino acid mutation. **(D)** 2019-nCoV and other bat SARS CoVs spike protein overlay comparison chart. The results show that S1 inserted into IB3 and S2 inserted into IB4, making the two regions have an outwardly extending loop structure (yellow is 2019-nCoV and blue is bat SARS CoV). CoV: Coronavirus; IB: Insert box; RBD: Receptor binding domain.

Although some amino acids were inserted in two positions of nsp3 in orf1ab [23], the insertion sites were in the nsp3b and nsp3c regions, which are mainly related to the binding reaction of nucleic acids. Because the insertion sites are not in the nsp3d region that contain PLpro (Figure 3A), the inserted sequence has little effect on the structure and function of PLpro. However, the two transmembrane domains contained in nsp3 are localized on nsp3b and nsp3c [24]. It may affect the localization of the nsp3 protein on the endoplasmic reticulum membrane [25]. The results of S protein sequence comparison showed the largest differences among all proteins. In the RBD site [26], three of the six key amino acid residues that interact with ACE2 have been changed. Pro^470^, Tyr^484^ and Thr^487^ are converted to Glu, Gln and Asn, respectively (Figure 3B). It is worth mentioning that the 470th amino acid was changed from nonpolar to polar amino acid. By analyzing the surface properties of the RBD structure of 2019-nCoV and bat SARS CoV (PDB ID: 6acc), we found that the RBD region polarity of the 2019-nCoV was more dense than the bat SARS CoV after mutation (Figure 3C). At the same time, four insert boxes (IBs; 1–4) were inserted into the N-terminus and S2 region of S protein in 2019-nCoV (Figure 3B). We selected the 2019-nCoV S protein with a low degree of homology comparison and compared it with the S protein of bat SARS CoV [27]. It was found that the insertion of IB3 increased the lateral expansion area of the S1 portion of the 2019-nCoV S protein, and a loop structure is extended at the overlap with the bat SARS CoV. The insertion of IB4 also adds a loop structure to the envelope region of S2 (Figure 3D), and the loop structure of proteins is often closely related to the structure and function of proteins [28,29].

### Back-mutating mutant amino acids to study the functional change of RBD of S protein

**Figure 4.**
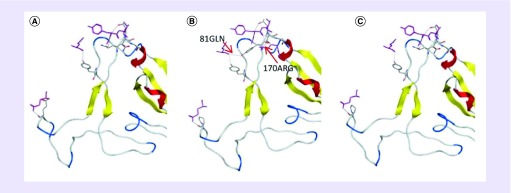
Amino acid back-mutation studies in the receptor binding domain of 2019-nCoV. **(A)** Result of interaction between 2019-nCoV and ACE2 before mutation. Purple is ACE2 amino acid residues. **(B)** Back mutation of three key amino acid residues interacting with ACE2 in receptor binding domain (RBD) of 2019-nCoV, and then docking with ACE2. It was found that based on the original hydrogen bond, Arg^170^ of ACE2 and Thr^486^ of RBD added a new hydrogen bond. Gln^81^ of ACE2 forms hydrogen bonds with Tyr^484^ while forming hydrogen bonds with Tyr^435^ of RBD (red arrow). **(C)** Back mutated the altered prolines in RBD of 2019-nCoV, then docked it with ACE2, and found that the binding ability of 2019-nCoV to ACE2 did not change significantly.

In order to study the effect of interactional amino acid changes in 2019-nCoV–ACE2 binding region RBD, we mutated the changed three amino acid residues (Glu^470^, Gln^484^ and Asn^487^) within the RBD structure back to the original amino acids. Then new, predicted structure is used to analyze the interaction between RBD and ACE2. We found that based on the original hydrogen bond, Arg^170^ of ACE2 and Thr^486^ of RBD added a new hydrogen bond. Gln^81^ of ACE2 forms hydrogen bonds with Tyr^484^ while forming hydrogen bonds with Tyr^435^ of RBD (Figure 4A & B). It is suggested that the mutation of three amino acid residues in RBD may weaken the 2019-nCoV interaction with ACE2. At the same time, we found that compared with ordinary bat SARS CoV, the four in the five prolines (Pro^458^, Pro^461^, Pro^465^, Pro^468^ and Pro^470^) of 2019-nCoV RBD were replaced with other amino acids (Figure 3B), so we changed the four amino acid residues to the original proline. The interaction between RBD and ACE2 was also analyzed to study the impact of this change on 2019-nCoV, but the results showed that the replacement of prolines has little effect on the interaction between 2019-nCoV and ACE2 (Figure 4A & C).

## Discussion

Through homology alignment, we elaborated the sequence differences of each key protein between 2019-nCoV and other bat SARS CoVs, and analyzed whether the new sequence changes in 2019-nCoV affected the function of each key protein. It was found that the sequence and protein structure of the structural proteins E, M and N of 2019-nCoV are basically consistent with that of bat SARS CoV. Considering that the structure determines the function, we believe that these three proteins should not be mutated. Although the orf1ab region has two large changes in the sequence, these changed positions are in nsp3b and nsp3c, not in the PLpro and 3CL hydrolase regions. Therefore, it has little effect on the two proteins that play a key role in the virus replication process. At the same time, we give examples of its application in the screening of peptide drugs after predicting the structure of PLpro. Among all proteins, the S protein has the largest variation, and most of the changes are located in the S1 region that interacts with ACE2. We also predicted two possible conformational changes of the S protein. They are similar to the changes of bat SARS CoV in the process of binding with ACE2, which suggests that the interaction mechanism between 2019-nCoV and ACE2 may be the same as bat SARS CoV. Based on the amino acid sequence and protein structure alignment, we found that the periphery of the S1 region is more extended than the general bat SARS CoVs. As the most direct structure with the outside world, this may eventually affect its binding to the receptor or its adsorption to objects. The three amino acids that interact with ACE2 are altered in the RBD of 2019-nCoV. By analyzing the surface properties of the protein, it was found that this change made the region more polar. In order to further study the effect of changed amino acids on the RBD, we back mutated these three amino acids and found that the mutated RBD structure has a stronger effect on ACE2. Because the RBD is more polar, and the number of hydrogen bonds it interacts with ACE2 is reduced, the strength of 2019-nCoV binding to ACE2 may be similar to common bat SARS CoV.

## Conclusion

The results presented in this manuscript demonstrate that the E, M and N protein of 2019-nCoV are not significantly different compared with the original bat SARS CoVs. The new fragment inserted in orf1ab has no effect on PLpro and 3CL hydrolase. Our predicted protein structure is highly reliable and can be used for drug development. We took the low-toxicity peptide library as an example, and successfully screened a tripeptide for potential drugs. For S proteins with large sequence differences, changes of key amino acid residues in RBD reduce the number of hydrogen bonds binding to ACE2. However, due to the extension of the loop structure in S1 and the increase of polarity, this may make the binding ability of 2019-nCoV and ACE2 similar to that of bat SARS CoV.

## Future perspective

From the appearance of the first 2019-nCoV infected patient to now, due to the convenience of modern transportation, its global impact far exceeded the SARS that occurred in Guangdong, China in 2003. CoV is an ssRNA structure, based on its instability, multiple virus mutations have been found in the past few years. However, because the experimental structure analysis of viral proteins requires time, it is difficult to meet the urgent need for drug development in emergencies. Although the virus mutates, it has a high degree of homology with the original bat SARS CoVs. The structure obtained by homology modeling has the potential to replace the crystal structure, and this will help to speed up drug development and facilitate structural and functional analysis of mutant viral proteins. It will play an important role in the future fight against different mutant viruses.

**Summary points**

- We elaborated the sequence and structure differences in each key protein of 2019-nCoV and other bat SARS coronaviruses (CoVs). We found no significant changes in envelope proteins, membrane proteins, nucleocapsid proteins and key proteases in open reading frame 1ab.
- We used the method of homology modeling to predict the structure of each key protein, then used molecular dynamics simulation to further process the predicted protein structure. On the basis of predicting a key protein structure, we also predicted two different state changes of S protein structure when it interacts with ACE2, and gave an example of the application of papain-like protease structure in peptide drug screening.
- We analyzed whether the new sequence changed in 2019-nCoV affected the function of each key protein. S protein has the largest variation among all proteins, and we used a virtual back-mutation method to study whether the new amino acid mutation of receptor binding domain of S protein in 2019-nCoV has an effect on the interaction between S protein and ACE2. Through a series of analyses, combined with the docking and simulation of S protein, we believe that the binding ability and mechanism of action between 2019-nCoV and ACE2 may be similar to that of bat SARS CoVs.
- This study combines bioinformatics tools and previous relevant experimental studies on the basis of viral sequences. It can overcome the problem of limited time and lack of experiments in the early stage of the disease, and has a good theoretical and practical basis.

## References

[1] Wang L, Su S, Bi Y, Wong G, Gao GF Bat-origin coronaviruses expand their host range to pigs. Trends Microbiol. 26(6), 466–470 (2018). 29680361 10.1016/j.tim.2018.03.001 PMC7119057

[2] Rha B Update: severe respiratory illness associated with a novel coronavirus–worldwide, 2012–2013. Am. J. Transplant. 13(6), 1606–1607 (2013). PMC4604826 23486385

[3] Li X, Zai J, Wang X, Li Y Potential of large ‘first generation’ human-to-human transmission of 2019-nCoV. J. Med. Virol. 92(4), 448–454 (2020). 31997390 10.1002/jmv.25693 PMC7166825

[4] Carlos WG, Dela Cruz CS, Cao B, Pasnick S, Jamil S Novel Wuhan (2019-nCoV) coronavirus. Am. J. Respir. Crit. Care Med. 201(4), p7–p8 (2020). 32004066 10.1164/rccm.2014P7

[5] Wang W, Tang J, Wei F Updated understanding of the outbreak of 2019 novel coronavirus (2019-nCoV) in Wuhan, China. J. Med. Virol. 92(4), 441–447 (2020). 31994742 10.1002/jmv.25689 PMC7167192

[6] Yoo JH The fight against the 2019-nCoV outbreak: an Arduous March has just begun. J. Korean Med. Sci. 35(4), e56 (2020). 31997618 10.3346/jkms.2020.35.e56 PMC6995816

[7] Eurosurveillance Editorial T. Note from the editors: novel coronavirus (2019-nCoV). EuroSurveill. 25(3), 2001231 (2020). 10.2807/1560-7917.ES.2020.25.3.2001231 PMC6988271 31992390

[8] Marra MA, Jones SJ, Astell CR The genome sequence of the SARS-associated coronavirus. Science 300(5624), 1399–1404 (2003). 12730501 10.1126/science.1085953

[9] Su S, Wong G, Shi W Epidemiology, genetic recombination and pathogenesis of coronaviruses. Trends Microbiol. 24(6), 490–502 (2016). 27012512 10.1016/j.tim.2016.03.003 PMC7125511

[10] Gui M, Song W, Zhou H Cryo-electron microscopy structures of the SARS-CoV spike glycoprotein reveal a prerequisite conformational state for receptor binding. Cell Res. 27(1), 119–129 (2017). 28008928 10.1038/cr.2016.152 PMC5223232

[11] Kirchdoerfer RN, Cottrell CA, Wang N Pre-fusion structure of a human coronavirus spike protein. Nature 531(7592), 118–121 (2016). 26935699 10.1038/nature17200 PMC4860016

[12] Li F Structure, function and evolution of coronavirus spike proteins. Annu. Rev. Virol. 3(1), 237–261 (2016). 27578435 10.1146/annurev-virology-110615-042301 PMC5457962

[13] Sievers F, Higgins DG Clustal Omega for making accurate alignments of many protein sequences. Protein Sci. 27(1), 135–145 (2018). 28884485 10.1002/pro.3290 PMC5734385

[14] Zhou P, Yang X-L, Wang X-G Discovery of a novel coronavirus associated with the recent pneumonia outbreak in humans and its potential bat origin. bioRxiv. (2020) (Epub ahead of print).

[15] Bordoli L, Kiefer F, Arnold K, Benkert P, Battey J, Schwede T Protein structure homology modeling using SWISS-MODEL workspace. Nat. Protoc. 4(1), 1–13 (2009). 19131951 10.1038/nprot.2008.197

[16] Kopp J, Schwede T The SWISS-MODEL repository: new features and functionalities. Nucleic Acids Res. 34(Suppl. 1), D315–D318 (2006). 16381875 10.1093/nar/gkj056 PMC1347419

[17] Schwede T, Kopp J, Guex N, Peitsch MC SWISS-MODEL: an automated protein homology-modeling server. Nucleic Acids Res. 31(13), 3381–3385 (2003). 12824332 10.1093/nar/gkg520 PMC168927

[18] Berry M, Fielding BC, Gamieldien J Potential broad spectrum inhibitors of the coronavirus 3CLpro: a virtual screening and structure-based drug design study. Viruses 7(12), 6642–6660 (2015). 26694449 10.3390/v7122963 PMC4690886

[19] Schoeman D, Fielding BC Coronavirus envelope protein: current knowledge. Virol. J. 16(1), 69 (2019). 31133031 10.1186/s12985-019-1182-0 PMC6537279

[20] Baez-Santos YM, St John SE, Mesecar AD The SARS-coronavirus papain-like protease: structure, function and inhibition by designed antiviral compounds. Antiviral Res. 115, 21–38 (2015). 25554382 10.1016/j.antiviral.2014.12.015 PMC5896749

[21] Wrapp D, Wang N, Corbett KS Cryo-EM structure of the 2019-nCoV spike in the prefusion conformation. Science 367(6483), 1260–1263 (2020). 32075877 10.1126/science.abb2507 PMC7164637

[22] Zhang L, Eiden LE Progress in regulatory peptide research. Ann. NY Acad. Sci. 1455(1), 5–11 (2019). 31646651 10.1111/nyas.14260 PMC13119615

[23] Lei J, Kusov Y, Hilgenfeld R Nsp3 of coronaviruses: structures and functions of a large multi-domain protein. Antiviral Res. 149, 58–74 (2018). 29128390 10.1016/j.antiviral.2017.11.001 PMC7113668

[24] Chatterjee A, Johnson MA, Serrano P Nuclear magnetic resonance structure shows that the severe acute respiratory syndrome coronavirus-unique domain contains a macrodomain fold. J. Virol. 83(4), 1823–1836 (2009). 19052085 10.1128/JVI.01781-08 PMC2643772

[25] Graham RL, Sparks JS, Eckerle LD, Sims AC, Denison MR SARS coronavirus replicase proteins in pathogenesis. Virus Res. 133(1), 88–100 (2008). 17397959 10.1016/j.virusres.2007.02.017 PMC2637536

[26] Li F, Li W, Farzan M, Harrison SC Structure of SARS coronavirus spike receptor-binding domain complexed with receptor. Science 309(5742), 1864–1868 (2005). 16166518 10.1126/science.1116480

[27] Kaplan W, Littlejohn TG Swiss-PDB viewer (deep view). Brief Bioinform. 2(2), 195–197 (2001). 11465736 10.1093/bib/2.2.195

[28] Fiser A, Do RK, Sali A Modeling of loops in protein structures. Protein Sci. 9(9), 1753–1773 (2000). 11045621 10.1110/ps.9.9.1753 PMC2144714

[29] Jiang H, Blouin C Ab initio construction of all-atom loop conformations. J. Mol. Model. 12(2), 221–228 (2006). 16247602 10.1007/s00894-005-0030-x

